# A tyrosine–tryptophan dyad and radical-based charge transfer in a ribonucleotide reductase-inspired maquette

**DOI:** 10.1038/ncomms10010

**Published:** 2015-12-02

**Authors:** Cynthia V. Pagba, Tyler G. McCaslin, Gianluigi Veglia, Fernando Porcelli, Jiby Yohannan, Zhanjun Guo, Miranda McDaniel, Bridgette A. Barry

**Affiliations:** 1 School of Chemistry and Biochemistry, Georgia Institute of Technology, Atlanta, Georgia 30332, USA; 2 Parker H. Petit Institute of Bioengineering and Bioscience, Georgia Institute of Technology, Atlanta, Georgia 30332, USA; 3 Department of Biochemistry, Biophysics and Molecular Biology, University of Minnesota, Minneapolis, Minnesota 55455, USA; 4 Department of Chemistry, University of Minnesota, Minneapolis, Minnesota 55455, USA; 5 Department for Innovation in Biological, Agro-food and Forest Systems, University of Tuscia, Viterbo 01100, Italy

## Abstract

In class 1a ribonucleotide reductase (RNR), a substrate-based radical is generated in the α2 subunit by long-distance electron transfer involving an essential tyrosyl radical (Y122O·) in the β2 subunit. The conserved W48 β2 is ∼10 Å from Y122OH; mutations at W48 inactivate RNR. Here, we design a beta hairpin peptide, which contains such an interacting tyrosine–tryptophan dyad. The NMR structure of the peptide establishes that there is no direct hydrogen bond between the phenol and the indole rings. However, electronic coupling between the tyrosine and tryptophan occurs in the peptide. In addition, downshifted ultraviolet resonance Raman (UVRR) frequencies are observed for the radical state, reproducing spectral downshifts observed for β2. The frequency downshifts of the ring and CO bands are consistent with charge transfer from YO· to W or another residue. Such a charge transfer mechanism implies a role for the β2 Y-W dyad in electron transfer.

## Abstract (web summary)

Tyrosine-tryptophan dyads are known to mediate electron transfer reactions in a range of different proteins. Here, the authors study a beta hairpin peptide, probing the tyrosine-tryptophan interaction and showing no hydrogen bonding but rather charge transfer between the tyrosyl radical and tryptophan'.

## Graphical abstract

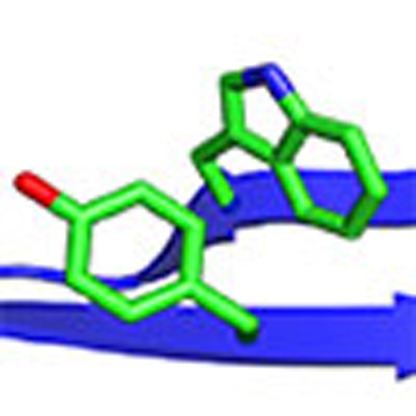

The aromatic amino-acid residues, tyrosine and tryptophan, mediate high potential electron transfer reactions in proteins^1^. For example, ribonucleotide reductase (RNR) employs a tyrosine-based charge relay to reduce ribonucleotides to deoxyribonucleotides^2^. The reaction is initiated by H atom abstraction at the substrate 3′-carbon; this process employs an active site cysteine radical in the α2 subunit. Class Ia RNRs, such as the *Escherichia coli* RNR, use a tyrosyl radical (Y122O·)-diferric cofactor in the β2 subunit to generate the cysteine radical. The charge transfer between Y122 and C439 occurs over 35 Å and is mediated by a conserved pathway of tyrosine side chains^3^. Y122O· is required for activity^4^ and is formed via oxygen-requiring reactions at the diferric cluster^4^,^5^,^6^.

Although there is no high-resolution structure of Y122O·-diferric β2, in structures of the met state (Y122OH-diferric state)^7^, Y122OH is buried in a hydrophobic environment and is ∼10 Å (Fig. 1a) from a surface-exposed tryptophan, β2 W48. This tryptophan is highly conserved in class 1a β2 sequences^8^. Mutations at W48 in *E. coli* β2 or at the homologous position, W103 in mouse β2 (Fig. 1b)^9^, inactivate RNR^8^,^10^,^11^,^12^. This residue has been shown to participate in cofactor assembly^10^. However, its role in proton-coupled electron transfer (PCET) has not been established^3^. The orientations of Y122OH and W48 in the crystal structure of the Y122OH-diferric state suggest the possibility of electronic, dipole–dipole interaction between the two side chains. In addition, the resonance Raman spectrum of Y122O· reveals downshifted CO and aromatic ring stretching frequencies. The low ring stretching frequency is not readily explained by existing model compounds^13^.

Despite the low sequence similarity between the *E. coli* and mammalian β2 subunits, the staggered T-shaped arrangement of tyrosine and tryptophan is similar when *E. coli*^7^, mouse^9^ and human p53 (ref. 14) β2 subunits are compared. Also, other metalloproteins containing redox active tyrosines, such as photosystem II (ref. 15) and galactose oxidase^16^, contain tyrosine–tryptophan dyads with a similar structure.

In this study, we designed a β hairpin peptide, Peptide M, to characterize the functional and spectroscopic consequences of such an interacting tyrosine–tryptophan pair. Peptide M is an 18-mer and is a variant of Peptide A (Fig. 1c), previously shown to form a β-hairpin (Fig. 1d)^17^,^18^. Peptide M (Fig. 1e) contains a single tyrosine and a single tryptophan, which form a dyad (Y5-W14), as established here by NMR, ultraviolet absorption and circular dichroism (CD) spectropolarography. The ultraviolet resonance Raman (UVRR) spectrum of Peptide M is unique and provides a framework in which to interpret the Raman spectra, previously reported for Y122O· in class 1a *E. coli* β2. Although there were earlier studies of (for example, see refs 19, 20) singlet states in model tyrosine–tryptophan peptides, we describe the first peptide in which interactions between the tyrosyl radical and the tryptophan sidechain are probed. The results are relevant to RNR and to other metalloproteins that employ redox-active tyrosines in high potential charge transfer pathways^21^,^22^.

## Results

### NMR studies

**Figure 1:**
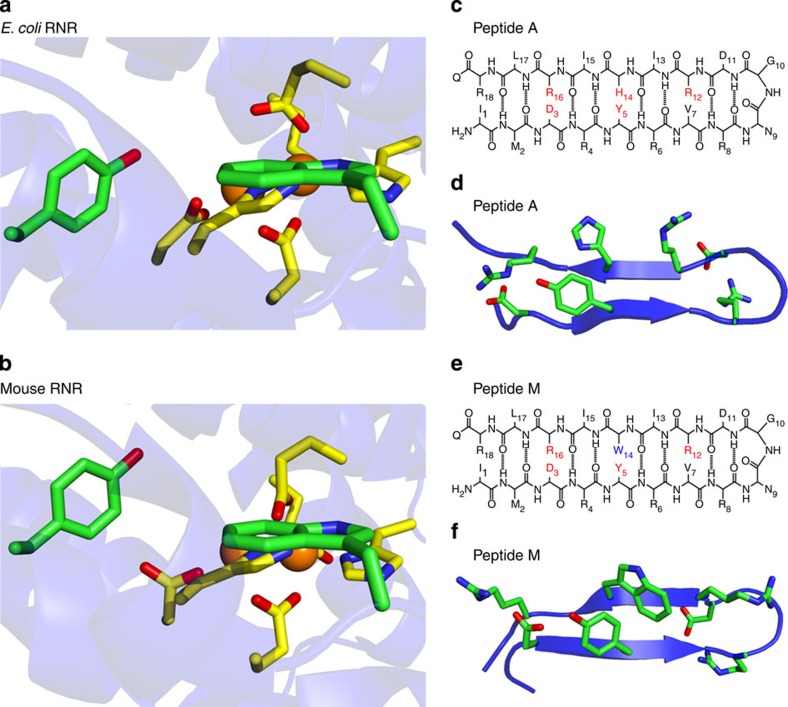
The environments of redox active tyrosine residues in class 1a met β2 subunits from *E. coli* and mouse RNR. The structures were generated with Pymol from 1MXR (*E. coli,* **a**) and 1W69 (mouse, **b**). In **a**, Y122 and W48 are shown, along with iron cluster ligands, D84, E115, H118, E204, E238 and H241 in *E. coli*. In **b**, Y177 and W103 are shown, along with iron ligands D139, E170, H173, E233, E267 and H270 in mouse. The distance between the phenolic oxygen and the indole nitrogen is 9.7 Å in *E. coli* and 9.9 Å in mouse. Primary sequences and NMR structures of Peptide A (**c**,**d**) and Peptide M (**e**,**f**). (**d**,**f**) The averaged, minimized NMR structures. The Peptide A structure was reported previously^17^, and the Peptide M structure is derived from this work. Structural analyses for the ensemble of 20 low energy NMR models are presented in Supplementary Tables 4 (Peptide M) and 5 (Peptide A) in the Supplementary Information, as well as side chain–side chain distances in the averaged, minimized structures.

**Figure 2:**
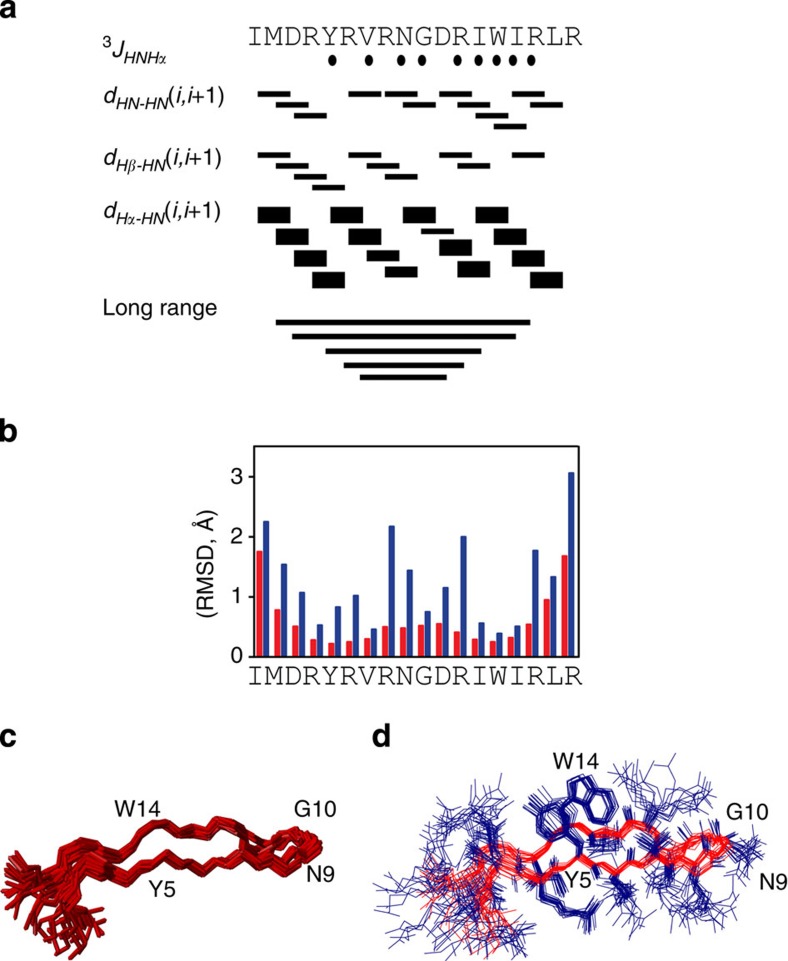
Summary of the NMR data on Peptide M. (**a**) Plot showing the short- and long-range ROEs for the backbone and the side chains, (**b**) root-mean-square deviations (RMSD) of the backbone atoms, (**c**) ensemble of 20 selected backbone conformers and (**d**) average structure of Peptide M. Resonance assignments were carried out using a combination of 2D [^1^H–^1^H]-TOCSY and [^1^H–^1^H]-ROESY experiments. All of the resonances were assigned, and 159 ROESY connectivities were detected. See Methods for more information, and the Supplementary Information for a table of ROE values used in calculations.

Peptide M is a variant of β-hairpin, Peptide A (Fig. 1c,d), in which H14 is replaced with W (Fig. 1e,f). For Peptide A, the NMR structure has shown that the peptide is β hairpin, that H14 has a pi–pi cross-strand interaction with Y5, and that Y5 is hydrogen bonded to R16 (ref. 17). To assess the structure of Peptide M in solution, we performed NMR experiments at pH ∼5. The NMR data are summarized in Fig. 2 and Supplementary Tables 1–3 in the Supplementary Information. The rotating frame Overhauser enhancement (ROE) pattern (Fig. 2a) with several (*i, i+1*) connectivities and ^*3*^*J*_*Hα-HN*_ coupling constants greater than 7 Hz for residues 5, 7, 10, 12, 13, 15 and 16 are typical of β strands. Also, several long-range dipolar contacts were observed between residues 2–17, 3–16, 5–14 and 6–13, supporting the β sheet fold of the peptide (Supplementary Fig. 1). The NMR data resulted in an ensemble of 20 selected conformers (Fig. 2c) with a root-mean-square deviation of 0.59±0.17 and 1.68±0.31 Å for backbone and side chains heavy atoms. The average structure (Figs 1f and 2d) shows that the aromatic residues (Y5 and W14) are co-facially aligned, as supported by seven inter-residue contacts between the aromatics, including ROEs between the ɛ protons of Y5 and CH_2_β and ɛ of W14. These NMR experiments show that the tyrosine and tryptophan are ∼6 Å apart and exhibit a pi-stacked, staggered interaction, which is reminiscent of the orientation observed in the β2 dyad. In Peptide M, tyrosine is not hydrogen bonded to tryptophan (distance greater than 5.9 Å in all models).

Seven of the twenty lowest energy conformers of the Peptide M ensemble display distances consistent with a hydrogen bond between the hydroxyl group of Y5 and NH of R16 (distance less than 3.0 Å, Supplementary Table 4). In Peptide A, Y5 is predicted to hydrogen bond as a proton acceptor to R16NH (distance less than 3 Å) in 6 of the 20 models and as a proton acceptor to R16NɛH in 4 of the 20 models (Supplementary Table 5). In the averaged, minimized structure of Peptide A, tyrosine is predicted to hydrogen bond to R16NɛH (Supplementary Table 5 and Fig. 1d). These results indicate that there is some probability of a hydrogen-bonding interaction between Y5 and R16 in both peptides. The dynamic nature of the peptide enables the formation of several stable as well as transient hydrogen bonds. Nonetheless, these residues are exposed to the bulk solvent, and intramolecular hydrogen bonds compete with formation of hydrogen bonds to water.

### Circular dichroism

**Figure 3:**
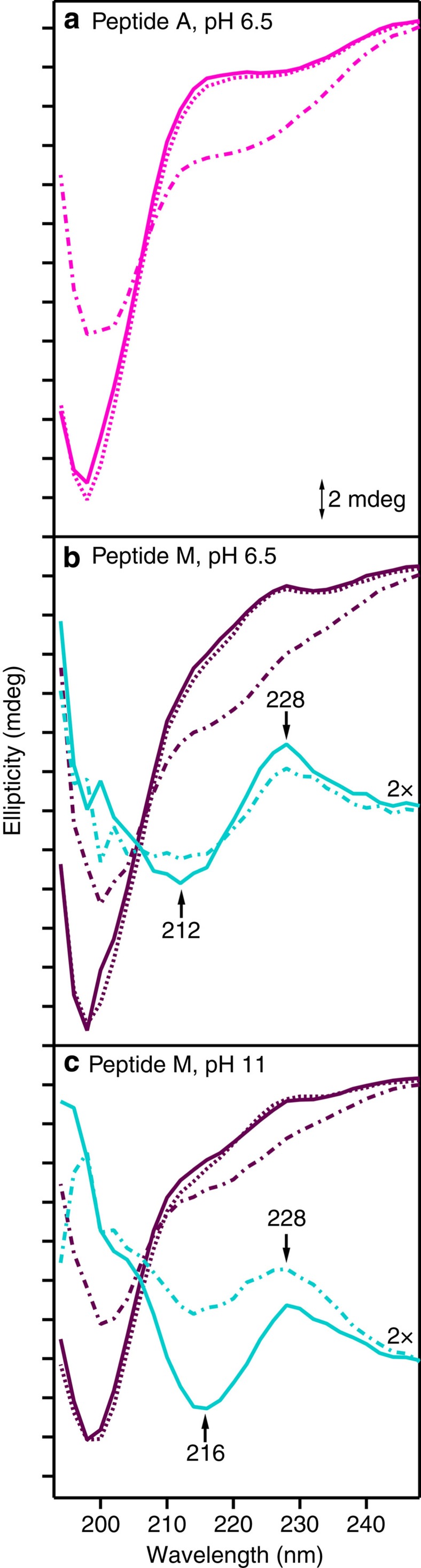
CD spectra of Peptide A and Peptide M. Data were acquired from Peptide A, pH 6.5 (**a**, pink) Peptide M, pH 6.5 (**b**, purple) and Peptide M, pH 11 (**c**, purple). The spectra were obtained at 20 °C (solid line, pre-melt), 80 °C (dot–dashed line) or at 20 °C (dashed line, post-melt). In **b**, difference CD spectra are shown in cyan, corresponding to Peptide M pH 6.5 (**b**, purple)–minus–Peptide A (**a**, pink). In **c**, difference spectra are shown in cyan, corresponding to Peptide M pH 11 (**c**, purple)–minus–Peptide A (**a**, pink). Data were obtained at 20 °C (**b**,**c**, cyan, solid line, pre-melt) or at 80 °C (**b**,**c**, dot–dashed line). The analyte concentration was 200 μM, and the buffer contained 5 mM MES, pH 6.5 (**a**,**b**) or 5 mM borate, pH 11 (C). The spectra were averaged from three independent measurements. The tick marks denote 2 mdeg. Difference CD spectra (cyan) in **b**,**c** are multiplied by a factor of 2 for presentation purposes.

Figure 3b,c presents CD data acquired from Peptide M. The characteristic β-hairpin CD signal has maximum negative ellipticity at 198 nm and arises from the n–pi* transition of the amide backbone^17^,^18^. Similar to Peptide A (Fig. 3a), Peptide M exhibits this characteristic β-hairpin band and reversibly folds both at pH 6.5 and 11. As shown, spectra obtained before (solid line) and after (dashed line) a thermal melt (80 °C, dot–dashed line) exhibit the characteristic negative 198 nm band of the β-hairpin. At both pH values, spectra obtained pre-melt (purple, solid line) and post-melt (purple, dashed line) are similar. Further, β-hairpin signal is lost at 80 °C (purple, dot–dashed line), consistent with thermal melting. These experiments establish that Peptide M has a stable, β-hairpin fold.

### Ultraviolet–visible absorption spectra

**Figure 4:**
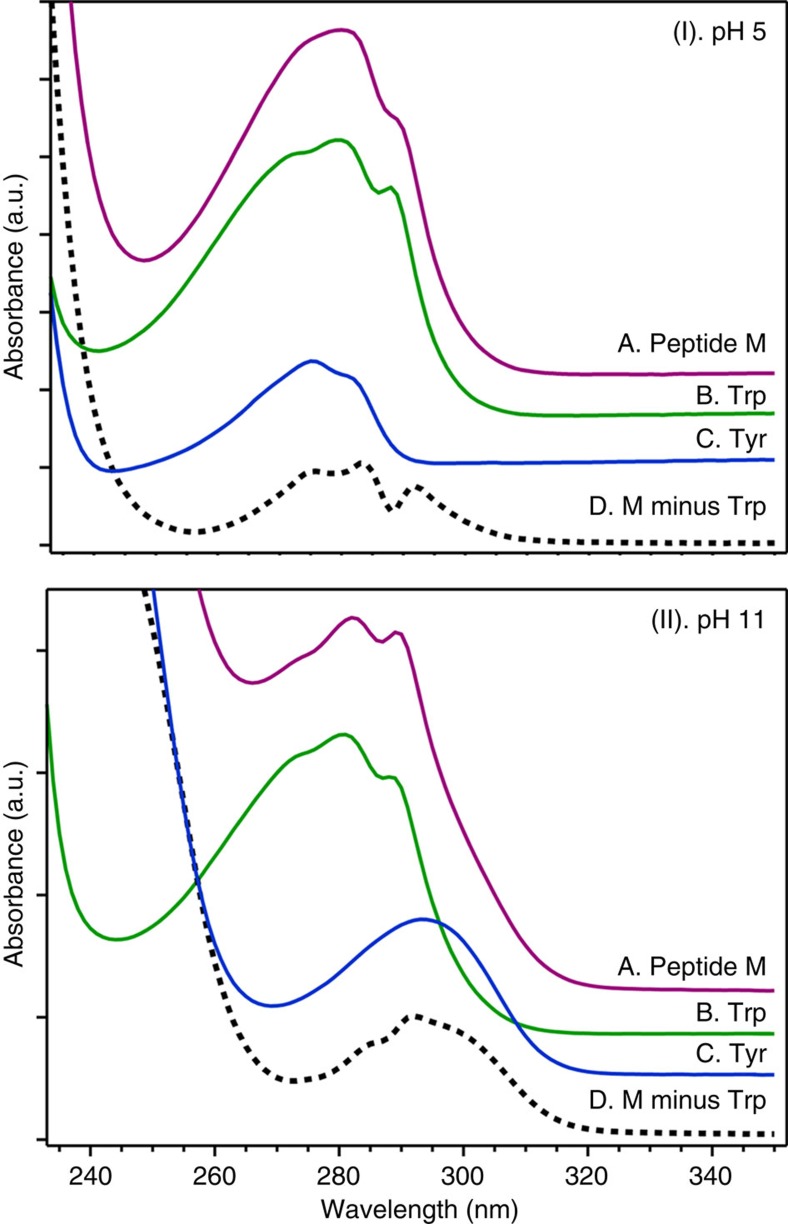
Ultraviolet absorption spectra of Peptide M and model compounds. Data were acquired from Peptide M (purple), tyrosine (Tyr; blue) and tryptophan (Trp; green) at pH 5 (I) and at pH 11 (II). The black dashed trace (D) was obtained by subtracting the tryptophan spectrum from that of Peptide M. The analyte concentration was 100 μM, and the buffer contained 5 mM acetate, pH 5 (I) or 5 mM borate, pH 11 (II). The spectra were averaged from two independent measurements. The tick marks denote 0.1 absorbance unit.

Figure 4a presents the ultraviolet absorption spectrum of Peptide M at pH 5 (I, purple) and pH 11 (II, purple). In the 200- to 300-nm region, the spectra of tyrosine (C) and tryptophan (B) are dominated by pi–pi* transitions of the phenol and indole rings. As expected, Peptide M (A) exhibits absorption bands similar to those of tyrosine and tryptophan. However, the Peptide M tyrosine spectrum is perturbed compared with solution models. Subtraction (D) of the aqueous tryptophan spectrum from that of Peptide M reveals a red-shift, both at pH 5 and 11 (I and II, black dashed line). Note that subtraction of the tyrosine spectrum from that of Peptide M yields spectra that resemble tryptophan (Supplementary Fig. 2, grey dotted line), with some minor perturbation, except for the region below 260 nm, which is the onset of the peptide bond absorption. Previous gas phase and computational studies of indole-benzene, indole-pyridine and indole-imidazole heterodimers have shown a stabilization of the S_0_ state, attributed to NH hydrogen bonding, NH– and CH–pi interactions and pi–pi interactions in the dimers^23^,^24^,^25^. Thus, the red shift of the tyrosine ultraviolet spectrum in Peptide M is attributable to the close proximity of the cross-strand Y5 and W14 to form a Y5-W14 dyad.

### Excitonic splitting

A through-space aromatic–aromatic interaction between two pi–pi* transitions with similar energies can result in excitonic splitting, which is detectable in the CD spectrum^26^. The red shift of the Peptide M tyrosine spectrum led us to search for characteristic exciton splitting in the CD spectra of this peptide. Examination of Fig. 3b (pH 6.5) and Fig. 3c (pH 11) showed a differential band, which is superimposed on the β strand 198 nm signal. Peptide A, which lacks the Y5-W14 dyad, did not exhibit this feature (Fig. 3a).

This excitonic feature is clearly seen when the CD spectrum of Peptide A is subtracted from that of Peptide M, revealing the characteristic excitonic splitting with a negative feature at 212–216 nm and a positive component at 228 nm. The amplitude of this excitonic couplet signal decreased at 80 °C, indicating sensitivity to thermal melting of the peptide. Excitonic coupling leads to the splitting of the excited state into two components because of delocalization over the two monomers. Previous studies of PagP have identified an excitonic couplet (negative, ∼225 nm; positive 230 nm) between Trp66 and Tyr26 (ref. 27). Such an excitonic splitting between the ^1^B_b_ band of tryptophan and the ^1^L_a_ band of tyrosine can occur only if the indole and phenol side chains are in close proximity and fulfill geometric restraints on the orientations of the two transition moments. We conclude that the tryptophan and tyrosine in Peptide M form an excitonically interacting, aromatic pair via a dipole–dipole interaction.

### Differential pulse voltammetry (DPV)

To characterize the properties of the dyad, we performed DPV, and the results are presented in Supplementary Fig. 3. The peak potential of tyrosine exhibits linear dependence on pH below the pK_a_ of the phenolic oxygen (10)^28^. Using DPV, the peak potentials of tyrosine (blue) were determined to be 0.99±0.01 and 0.65±0.01 V at pH 5 and 11, respectively. The voltammograms acquired from tyrosine were irreversible, as previously reported, because of a contribution from competing reactions^28^. However, these peak potentials agree with reported midpoint potentials, implying that any correction factor is small^17^,^28^,^29^. Y5 in Peptide A (pink) gave peak potentials of 1.00±0.01 V (pH 5) and 0.71±0.02 V (pH 11) versus normal hydrogen electrode (NHE), similar to peak potentials of aqueous tyrosine and to values previously reported for this peptide^17^.

The peak potentials of Peptide M, aqueous tryptophan and an aqueous mixture of tyrosine and tryptophan (Supplementary Fig. 3A,B) were similar both at pH 5 and pH 11, given the standard deviation of the measurements. The values obtained at pH 5 and 11 for Peptide M (1.02±0.02 and 0.72±0.01 V versus NHE, respectively) were in good agreement with values previously reported for the midpoint potential of tryptophan^28^,^29^. We conclude that the electronic interaction between tryptophan and tyrosine in Peptide M does not have a large effect on the peak potential of the amino-acid side chain. This implies that stabilization of the singlet and the radical state are similar in Peptide M, suggesting that Y5 interacts with W14 in both states.

### Electron paramagnetic resonance (EPR) spectroscopy

Next, we sought to examine the radical state of Y5 in order to establish whether tyrosyl radical structure is altered in Peptide M. To examine the radical state, ultraviolet photolysis and EPR spectroscopy can be performed. However, the breadth of the X-band EPR spectra precludes the detection of small differences in radical structure. This is illustrated in Supplementary Fig. 4, in which photolysis at 266 nm was used to generate both tyrosyl (Supplementary Fig. 4D) and tryptophan (Supplementary Fig. 4C) radicals in frozen solutions or powders (160 K) of the isolated amino acids. The EPR of the tyrosyl and tryptophan radicals agree in lineshape and *g* value with earlier reports^30^,^31^. Ultraviolet photolysis of Peptide M (Supplementary Fig. 4B) gave an EPR lineshape similar to the spectrum of an aqueous mixture of tyrosine and tryptophan (Supplementary Fig. 4C) and of Peptide A (Supplementary Fig. 4A). However, small changes due to the environment are not detectable. Note that this procedure does not cause significant modification of the samples as assessed by the ultraviolet–visible absorption before and after photolysis (Supplementary Fig. 5).

### Ultraviolet resonance Raman studies

To more incisively probe the structure of the tyrosyl radical and singlet state, UVRR spectra were acquired at pH 11 using 244 nm excitation. Previous work has shown that tyrosyl radical can be generated by 244 nm, continuous illumination of aqueous tyrosine at room temperature^13^,^32^. This procedure does not result in modification of the peptide, as assessed by mass spectrometry. Raman excitation at 244 nm is specific for the tyrosyl radical and the tyrosine singlet, because of resonance enhancement of the tyrosine electronic transition relative to that of tryptophan. The Raman bands due to tyrosyl radical and tyrosine singlet state are highlighted in difference spectra (high power scan minus low power scan). The positive bands correspond to the vibrational modes of the radical, whereas the negative bands correspond to the singlet vibrational modes.

**Figure 5:**
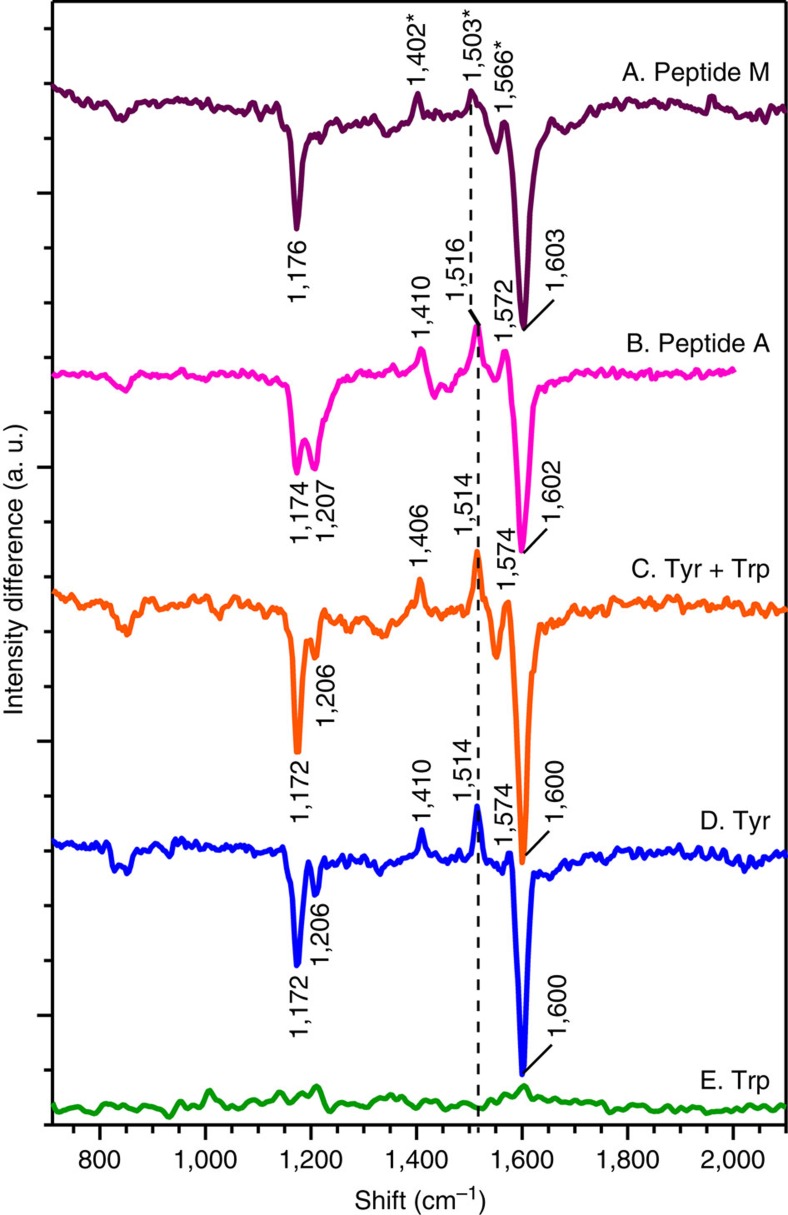
UVRR difference spectra derived from Peptide M, Peptide A and model compounds. Data were acquired from Peptide M (A), Peptide A (B), a tyrosine-tryptophan solution (C), a tyrosine solution (D) or a tryptophan solution (E). The analyte concentration was 1 mM, and the buffer was 5 mM borate, pH 11. The difference spectrum was obtained by subtracting an averaged low-power scan (340 μW) from an averaged high-power scan (3.4 mW). The tick marks denote 50 intensity units. The data were averaged from at least two independent measurements. The asterisks in A denote unique frequencies of the YO·-W dyad.

The UVRR difference spectrum obtained from aqueous tyrosine is shown in Fig. 5d. Positive bands at 1,410, 1,514 and 1,574 cm^−1^ are assigned to ring stretching (Y19a), C–O stretching (Y7a) and C–C ring (Y8a) stretching modes of the radical, respectively (Table 1). The negative bands at 1,172, 1,206 and 1,600 cm^−1^ arise from the CH bend (Y9a), ring C–CH2 (Y7a) and a ring Y8a mode of the singlet state^13^,^32^. As expected, 244 nm excitation of aqueous tryptophan did not produce a Raman difference spectrum (Fig. 5e). The difference spectrum of an aqueous tyrosine–tryptophan mixture (Fig. 5c) was indistinguishable (±2 cm^−1^) from that of tyrosine alone, again showing the specificity of this Raman probe wavelength for tyrosine.

Figure 5a presents UVRR difference spectrum of Peptide M. The frequencies and intensities of the singlet Raman bands were identifiable using data acquired from the tryptophan–tyrosine mixture and a tyrosine solution. A shift in the frequency of the singlet Y8a, from 1,600 cm^−1^ in tyrosine (Fig. 5d) to 1,603 cm^−1^ in Peptide M (Fig. 5a), may not be significant, relative to the uncertainty in peak position (±2 cm^−1^). The Y9a band is conformationally sensitive and exhibits a modest shift (4 cm^−1^) when Peptide M is compared with tyrosine.

In contrast, the frequencies of the positive radical bands were significantly shifted in Peptide M, compared with those derived from tyrosine, a solution of tyrosine and tryptophan, and Peptide A (Fig. 5b and Table 1)^13^,^32^. For example, the radical ring stretch (Y19a) shifts from 1,410 cm^−1^ in tyrosine to 1,402 cm^−1^ in Peptide M (Table 1). In addition, the radical CO stretch (Y7a) and the ring stretch (Y8a) bands were 1,514 and 1,574 cm^−1^ in the tyrosine model compound (Fig. 5d) but were 1,503 and 1,566 cm^−1^ in Peptide M (Fig. 5a, peaks labelled with asterisks). Radical frequencies in Peptide A were indistinguishable from that of tyrosine and were 1,410 (Y19a), 1,516 (Y7a) and 1,572 (Y8a) cm^−1^ (Table 1 and see also ref. 32). We conclude that substantial 8–11 cm^−1^ Raman shifts are diagnostic for an aromatic–aromatic interaction between the tyrosyl radical and a tryptophan side chain in a YO·-W dyad.

## Discussion

In β2 or other complex proteins, a detailed picture of interaction between Y and W is difficult to obtain, because of background signals from other tyrosines, tryptophans and the peptide backbone. *De novo* designed peptides provide structurally tractable backgrounds in which to elucidate function (for examples, see refs 29, 33, 34). For instance, studies of the β-hairpin, Peptide A, have demonstrated that the peptide environment accelerates the rate of electron and proton-coupled electron transfer^35^ and provide a model system for investigating orthogonal proton and electron transfer involving tyrosine and histidine^17^,^18^. Also, UVRR spectroscopy has shown that a reversible conformational change accompanies electron or proton-coupled electron transfer in Peptide A^32^.

To understand the spectroscopic and functional properties of a tyrosine–tryptophan dyad, we designed and synthesized a β-hairpin maquette, Peptide M, which contains a tyrosine–tryptophan cross-strand interacting pair. Peptide M forms a β-hairpin at pH 6.5 and 11 and can be reversibly folded and unfolded at both pH values. NMR experiments show that the tyrosine and tryptophan are ∼6 Å apart and exhibit a pi-stacked, staggered interaction, which is similar to the tyrosine/tryptophan orientation observed in β2. The ultraviolet and CD spectra of the peptide exhibit spectral shifts and splittings that are characteristic of S_0_ state stabilization and of a dipole–dipole-mediated electronic interaction between the indole and phenol groups, respectively. The peak potential and singlet Raman spectrum of Peptide M are not significantly perturbed, compared with the isolated amino acids or Peptide A. However, the Raman spectrum of the radical, Y5·-W14, dyad in Peptide M is distinct from that of tyrosine and Peptide A.

In model tyrosine, production of tyrosyl radical is associated with a dramatic upshift of the CO vibrational band (from ∼1,260 to 1,514 cm^−1^) and a downshift of the highest energy aromatic ring stretching mode (from ∼1,600 to 1,574 cm^−1^)^13^,^36^,^37^,^38^. The Y5·-W14 dyad in Peptide M (Fig. 5a) exhibits downshifted CO and ring stretching bands, relative to those of tyrosyl radical in aqueous solution (Fig. 5d) and Peptide A (Fig. 5b). Interestingly, downshifted CO and ring stretching frequencies have also been reported for Y122O·-W48 in *E. coli* β2 (refs 13, 36, 39). The CO band of the Y122O·-W48 dyad was detected at 1,498/1,499 cm^−1^ (refs 13, 36, 39), and the ring stretching mode was detected at 1,556 cm^−1^. The substantial downshifts observed in the Peptide M dyad suggest that the shifted Raman frequencies in *E. coli* β2 are due, at least in part, to an interaction between Y122O· and W48. The CO band has also been reported to be sensitive to hydrogen bonding and electrostatics^40^,^41^, but a factor that downshifts the frequency of the ring stretching bands has not been successfully modelled until this work (reviewed in ref. 42). Note that in the Peptide M structural ensemble calculated via NOE, there are no conformers showing the presence of an H-bond between Y5 and W14. However, seven of the lowest energy conformers show the presence of an H-bond between the side chains of Y5 and R16. Therefore, intramolecular hydrogen bonding does not provide an explanation for the downshifted Raman bands of Peptide M, when compared with Raman bands of Peptide A.

We attribute the downshift of the CO and ring frequencies of the tyrosyl radical to charge transfer from the tyrosyl radical in the Y·-W dyad. Precedent is established in previous studies of Old Yellow Enzyme, in which charge transfer between flavin cofactor and phenolic compounds resulted in shifts of phenol Raman bands^43^. Subtle changes in angle and distance are observed in the met state of *E. coli* and mouse β2; these changes in orientation could influence the amount of charge transfer. UVRR studies to detect both the ring and CO stretching mode in mammalian β2 will be useful in evaluating this possibility. Such subtle changes in orientation may be the foundation of the 4–8 cm^−1^ differences in band position, when RNR and Peptide M are compared. Note that the idea of conformational gating and tyrosyl radical movement has been proposed to explain the EPR and FTIR spectra of hydrogen peroxide-treated crystals^7^ and hydroxyurea-treated solution samples^38^, respectively. Therefore, the met Y122OH structure may not be predictive of all the interactions in the radical state.

In summary, Peptide M is the first reported model system that accounts for the downshifted CO and ring stretching vibrations of Y122O· in *E. coli* β2. The results presented here suggest that the *E. coli* β2 Y122O· radical interacts with W48. The decrease in vibrational frequencies for Y122O· is characteristic of an overall decrease in radical bond strength. This decrease can be associated with delocalization of electron density onto the W48 indole ring or onto another nearby site in the protein. Note that a small amount of charge transfer from the tyrosyl radical to another site would be difficult to detect with magnetic resonance techniques. However, this transfer of charge may be an important element in guiding electron transfer through the hydrogen-bonding network of β2. This charge transfer motif may also operate in other metalloproteins, which employ redox-active tyrosine residues.

## Methods

### Material

Peptides were synthesized by solid-state synthesis and were obtained from Genscript USA Inc. ^2^H_2_O and NaO^2^H were purchased from Cambridge Isotopes (99.9% D). When ^2^H_2_O was used, the p^2^H was adjusted to the uncorrected metre reading^44^. 2-(*N*-morpholino)ethanesulfonic acid (MES), *N*-cyclohexyl-3-aminopropanesulfonic acid (CAPS), 2-[4-(2-hydroxyethyl)piperazin-1-yl]ethanesulfonic acid (HEPES), tyrosine and tryptophan were purchased from Sigma-Aldrich. Sodium borate was purchased from Mallinckrodt Pharmaceuticals, and boric acid was purchased from J.T. Baker Avantor Performance Materials. Hexamine ruthenium (III) chloride was purchased from Strem Chemicals.

### NMR

The NMR samples were prepared by dissolving 1 mg of peptide in 350 μM sodium phosphate buffer (pH ∼5) containing 5% ^2^H_2_O. The spectra were acquired on a 700-MHz Bruker spectrometer equipped with a triple-resonance cryogenic probe. A [^1^H,^1^H]-total correlation spectroscopy (TOCSY) experiment with 70 ms mixing time and spectral widths of 7,122.5 Hz for both dimensions was utilized to assign all of the peptide resonances. MLEV17 with field strength of 9 kHz was used as a mixing sequence^45^. To determine the inter-nuclear distances, we utilized a series of [^1^H,^1^H]-rotating frame nuclear Overhauser effect spectroscopy (ROESY) experiments with spectral widths identical to the TOCSY experiments, and with a continuous wave mixing sequence using 100, 200, 250, 350 and 400 ms mixing times^46^. The NMR spectra were processed using NMRPipe^47^ and analysed using Sparky^48^ software packages. The spectra were referenced to the water resonance at 4.7 p.p.m. TOCSY and ROESY spectra were assigned using the standard approach described by Wuthrich^49^. Based on the build-up curve, ROESY cross-peaks at 350 ms were chosen for structure calculations. A total of 156 ROEs consisting of 65 intra-residue and 91 inter-residue correlations were used for structure determination. Peak intensities were converted into distances using the following classifications: strong (1.8–2.9 Å), medium (1.8–3.6 Å) and weak (1.8–6.0 Å). For residues with ^3^*J*_*NH-Ha*_>8.0 Hz φ, angles were restricted to the range of –160 to –80°. NMR conformers were calculated starting from an extended conformation of Peptide M and using a hybrid simulated annealing protocol available in the XPLOR-NIH package^50^. Briefly, calculations were carried out using an initial temperature of 1,000 K, 50,000 high temperature steps and 6,000 cooling steps with a step size of 5 fs decreasing the temperature from 1,000 to 100 K. The generated conformers were further minimized including the Lennard-Jones potential, with the Conjugated Gradients algorithm using an initial temperature of 300 K and 60,000 steps (step size 1 fs). A total of 50 conformers were generated; the 20 lowest energy conformers without NOE violations greater than 0.5 Å, no bond violations greater than 0.05 Å, and no bond violations greater than 4° were selected for further analysis. Measurements were conducted both at 278 and 298 K.

### Circular dichroism

A Jasco J-810 CD spectropolarimeter equipped with a Peltier-type cell was employed. Spectra were collected from 250 to 193 nm in 1 mm quartz cells. Eight accumulations per scan were averaged in three independent measurements for each of the conditions. Parameters used were: sensitivity, 100 mdeg; data pitch, 2 nm; scan speed, 50 nm min^−1^; response time, 1 s; bandwidth, 1 nm.

### Ultraviolet–visible spectroscopy

Ultraviolet absorption spectra were recorded on a Shimadzu UV-1700 spectrometer. The slit width was 1 nm, the resolution was 1 nm and the scan speed was 6.5 nm s^−1^. The spectra were averaged from two independent measurements.

### Ultraviolet resonance Raman

UVRR spectra of 1 mM samples were obtained at room temperature using a 244-nm probe beam generated from an intracavity frequency-doubled Argon ion laser (Cambridge LEXEL 95)^32^,^51^. Briefly, the probe beam was coupled to a Raman microscope system (Renishaw inVia) equipped with ultraviolet-coated, deep depletion charge-coupled device. Backscattering from the sample was collected by a 15 × ultraviolet (NA=0.32) objective (OFR division of Thorlabs, Inc.), assembled in a Leica Microsystems microscope. The spectral resolution was 6 cm^−1^, and the interval between the data points was 3.8 cm^−1^. The peak positions are reported to a precision of ±2 cm^−1^. To prevent photodegradation, samples were re-circulated using a peristaltic pump and a nozzle (∼120 μm inner diameter) to form a jet. The Raman difference spectrum was obtained by subtracting an averaged low power scan (340 μW) from an averaged high power scan (3.4 mW) using 244 nm beam. The data were averaged from at least two measurements on different samples. Mass spectrometry (ThermoFisher Scientific, LTQ Orbitrap XL, Electrospray ionization, Positive Ion Mode) before and after the Raman measurement gave the expected 1,195 *m*/*z* ratio for Peptide M, indicating that there was no significant oxidative modification of the peptide during the UVRR measurement.

## Additional information

**How to cite this article:** Pagba, C. V. *et al.* A tyrosine–tryptophan dyad and radical-based charge transfer in a ribonucleotide reductase-inspired maquette. *Nat. Commun.* 6:10010 doi: 10.1038/ncomms10010 (2015).

## Supplementary Material

Supplementary Information Supplementary Figures 1-5, Supplementary Tables 1-5 and Supplementary References

## Figures and Tables

**Table 1:** Vibrational frequencies (cm^−1^) and assignments for tyrosyl radical in Peptide M, other model compounds and *E. coli* **β**2 as defined by UVRR spectroscopy.

**Sample**	**RingY8a**	**CO Y7a**	**Ring Y19a**
Tyr*	1,574	1,514	1,410
Tyr+Trp*	1,574	1,514	1,406
Trp*	NO†	NO†	NO†
Peptide A*	1,572	1,516	1,410
Peptide M*	1,566	1,503	1,402
RNR‡	1,556	1,499	NO†

RNR, ribonucleotide reductase; Trp, tryptophan; Tyr, tyrosine; UVRR, ultraviolet resonance Raman.

^*^This work.

^†^NO, not observed.

^‡^Values from ref. 13.
