# Supplementary material for: A tyrosine–tryptophan dyad and radical-based charge transfer in a ribonucleotide reductase-inspired maquette
Source: Nat Commun. 2015 Dec 2;6:10010. doi: 10.1038/ncomms10010 (PMC4686667; doi:10.1038/ncomms10010)
Supplement: Supplementary Information — Supplementary Figures 1-5, Supplementary Tables 1-5 and Supplementary References [file ncomms10010-s1.pdf]

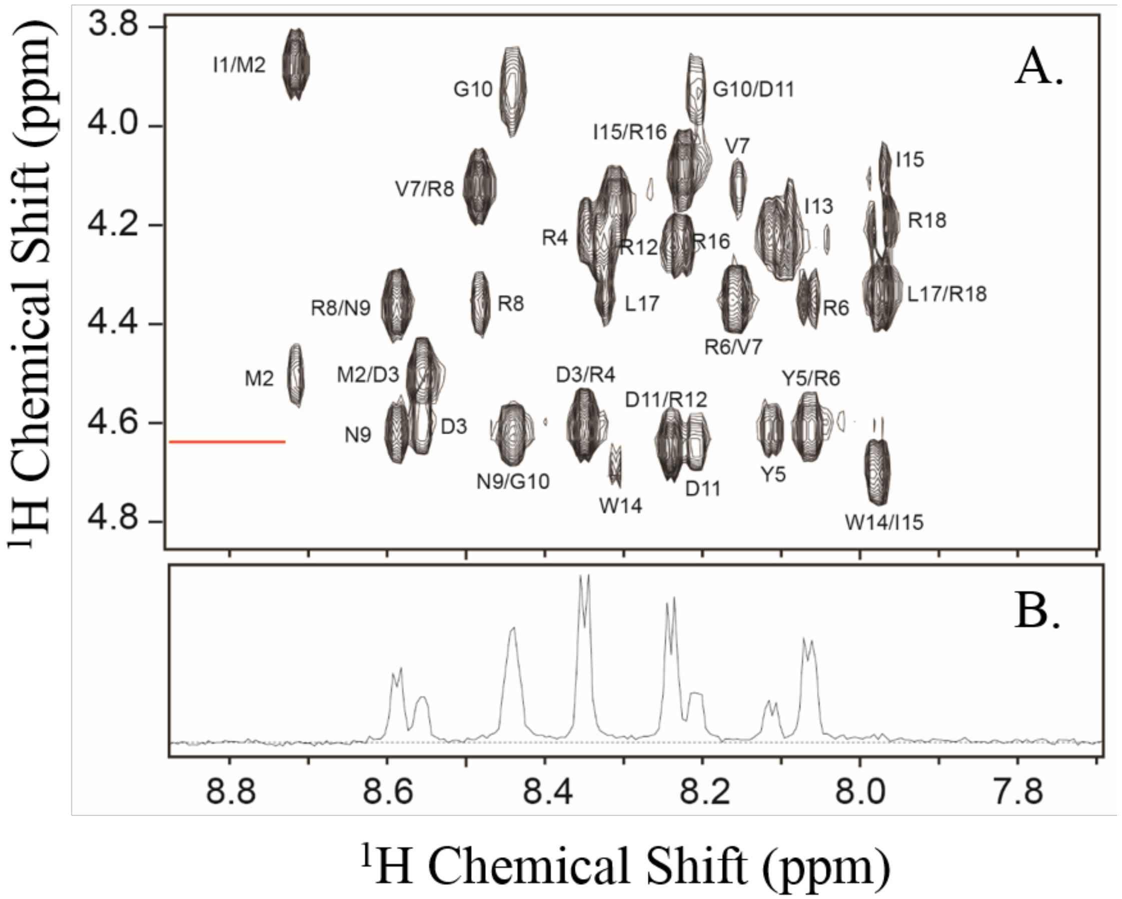

Supplementary Figure 1. NMR data. Selected region of [^{1}H,^{1}H] ROESY (A) and ^{1}H-NMR (B) spectra showing several long-range dipolar contacts between residues.

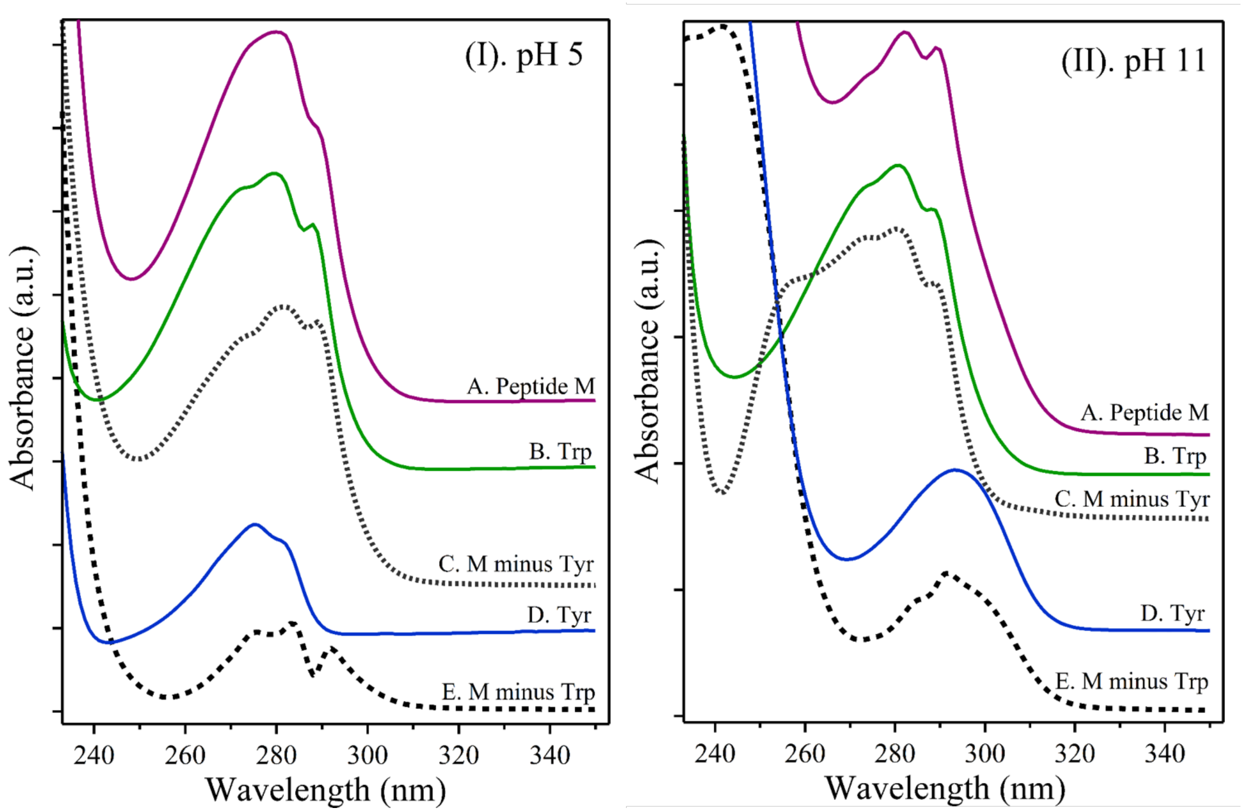

Supplementary Figure 2. UV absorption spectra. Data were derived from Peptide M (purple), tyrosine (blue) and tryptophan (green) at pH 5 (I) and at pH 11 (II). The gray dotted trace (C) was obtained by subtracting the tyrosine spectrum from that of Peptide M. The black dashed trace (E) was obtained by subtracting the tryptophan spectrum from that of Peptide M. The analyte concentration was 100 µM, and the buffer contained 5 mM acetate, pH 5 (I) or 5 mM borate, pH (11). The spectra were averaged from two independent measurements. The tick marks denote 0.1 absorbance unit.

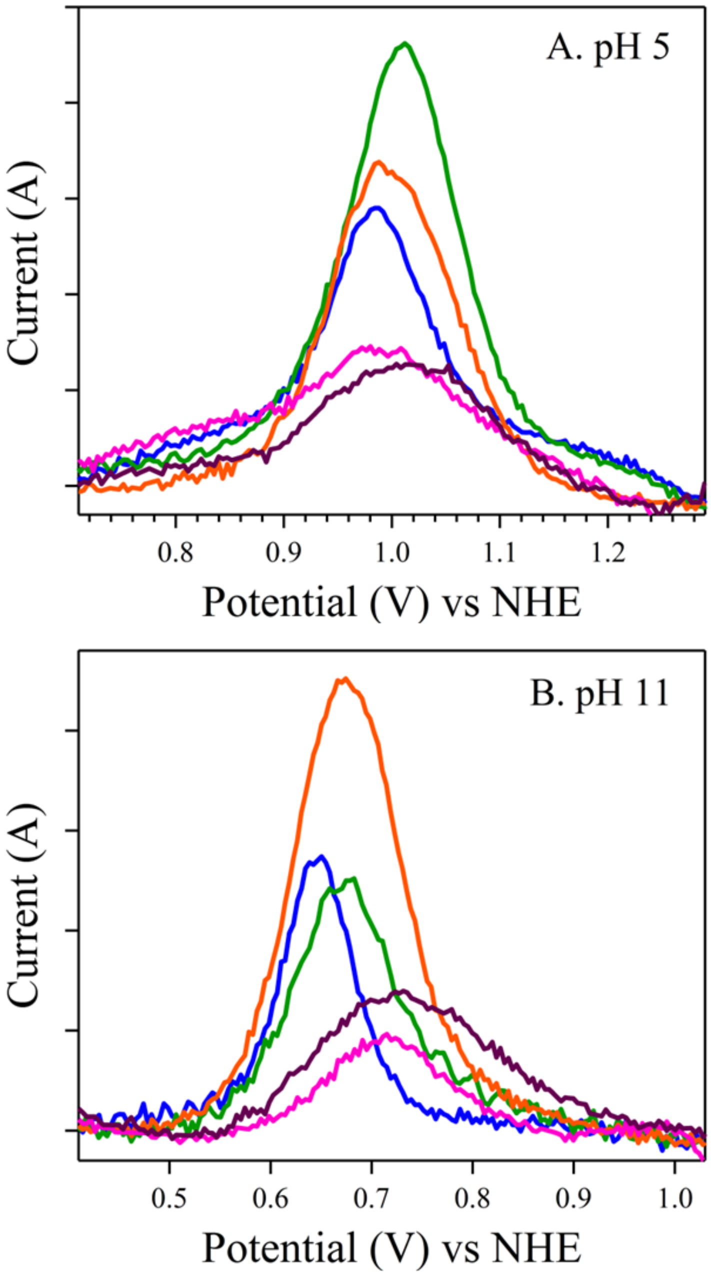

Supplementary Figure 3. DPV. Data were derived from Peptide A (pink), Peptide M (purple), a tyrosine-tryptophan solution (orange), a tyrosine solution (blue) and a tryptophan solution (green) at pH 5 (A) or at pH 11 (B). The data were baseline corrected for presentation purposes. The analyte concentration was 100 μM. At pH 5, the buffer was 5 mM acetate, 200 mM KCl (A). At pH 11, the buffer was 5 mM borate, 200 mM KCl (B). The data were averaged from three independent measurements for peptides and from nine independent measurements for amino acid analytes. Potentials are given versus the normal hydrogen electrode (NHE) by adding 0.22 V to the values measured using a 1 M KCl-filled Ag/AgCl reference electrode. The tick marks denote 1 x 10^{-7} units. Peptide A (pink) and Peptide M (purple) voltammagrams are multiplied by 2 for

clarity. DPV measurements were performed on a computer-controlled Princeton Applied Research 273A potentiostat. Experiments were conducted in an argon-sparged, three-electrode cell (CH Instruments, Austin, TX) equipped with a 3 mm glassy carbon working electrode, platinum wire counter electrode, and a Ag/AgCl reference electrode in 1 M KCl (E = 0.22 V (NHE)). Data were collected in increments of ΔE = 4 mV at a scan rate of 32 mV/s. The differential pulse amplitude was 25 mV. Data were fit to a polynomial baseline using PeakFit 4 (Systat Software Inc, San Jose, CA) and smoothed using CorrView3 (Scribner, Southern Pines, NC). Data on the amino acids were averaged in triplicate and data on the peptides were averages of nine trials. The peak potentials reported in the text were determined from the centroid of the data before baseline correction. Hexamine ruthenium (III) chloride (200 μM, 1 M KCl)[1] was used as standard in each trial and gave a peak potential of -0.200 ± 0.003 V versus Ag/AgCl (average of 20), as expected. In control experiments at pH 11, CAPS was used as a buffering agent. The substitution of the borate buffer by CAPS had no effect on the DPV measurement at pH 11.

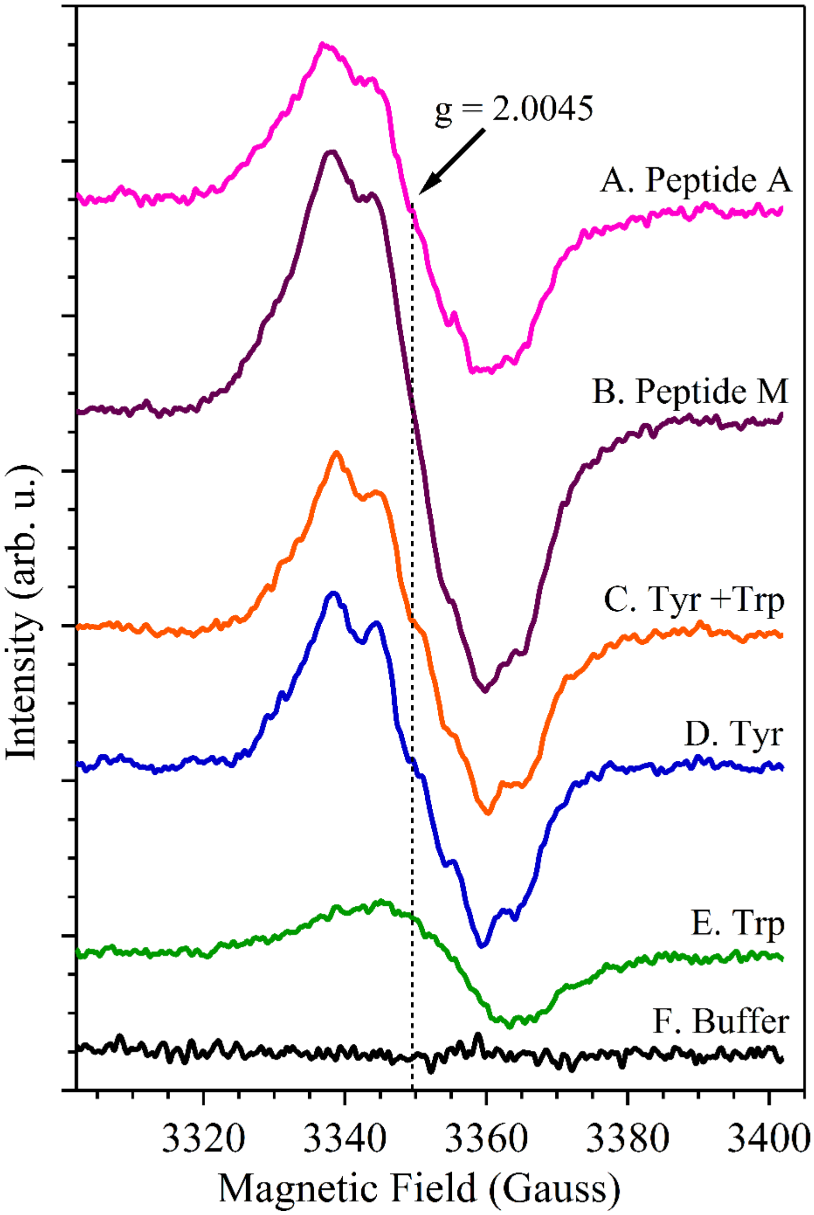

Supplementary Figure 4. X-band EPR spectra. Data were acquired from Peptide A (A), Peptide M (B), tyrosine + tryptophan solution (C), tyrosine solution (D) and tryptophan solution (E). The buffer blank is shown in (F). EPR spectra[2,3] were collected on a Bruker EMX spectrometer (Billerica, MA) at 160 K using the following conditions: microwave frequency, 9.2 GHz; microwave power, 200 µW; modulation amplitude, 1 G; modulation frequency, 100 kHz; scan time, 168 s; number of scans, 4; time constant, 655 ms. Radicals were generated using 50 flashes at 266 nm (50-60 mJ) generated by a Nd-YAG laser (Continuum Surelite III, Santa Clara, CA). The analyte concentration was 1 mM, and the buffer contained 10 mM borate, pH 11. The data were averaged from three independent measurements. The tick marks denote 500 units.

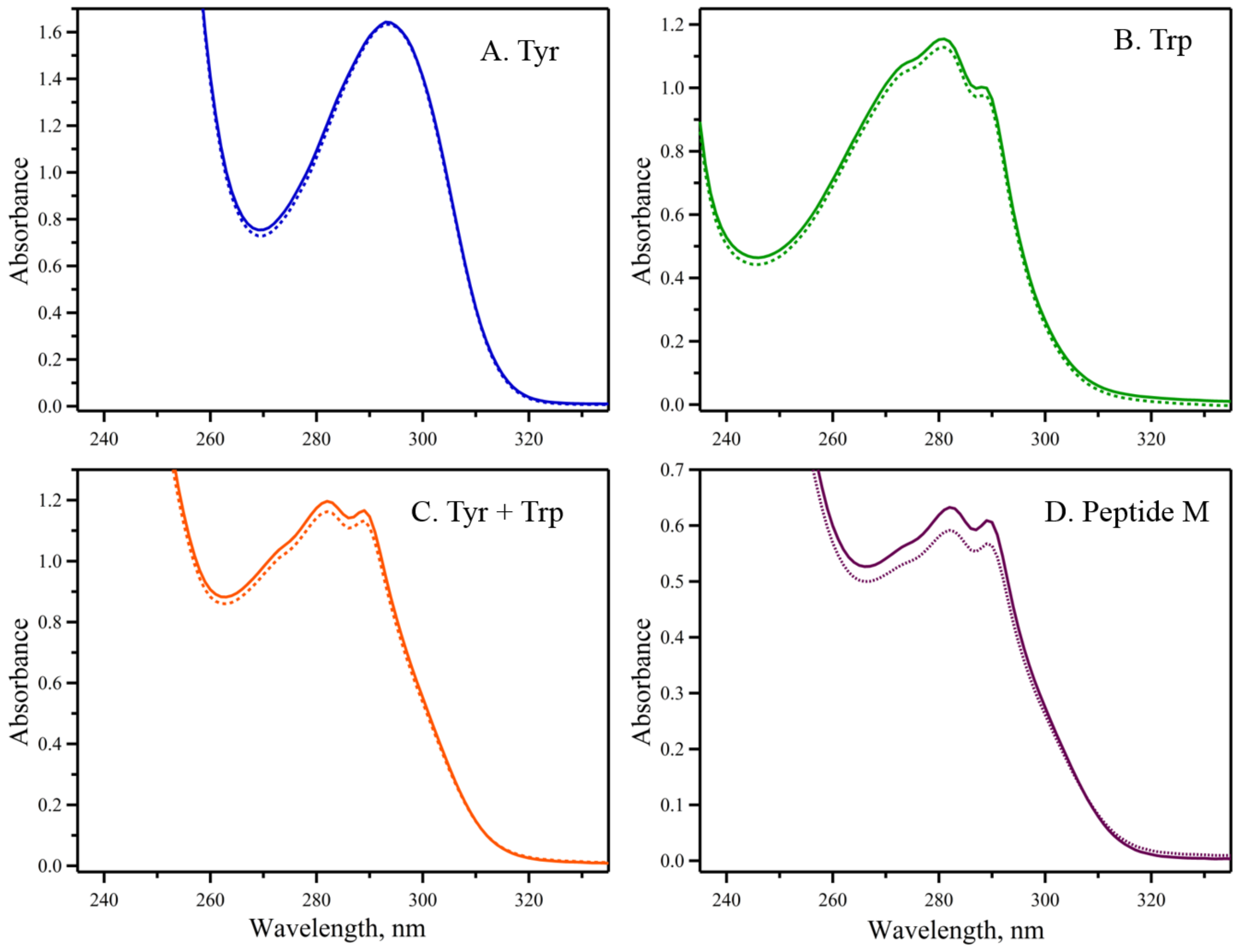

Supplementary Figure 5. UV-Vis spectra. Data acquired from tyrosine (A), tryptophan (B), tyrosine-tryptophan solution (C) and Peptide M (D) before (solid line) and after (dashed-line) EPR measurements. The solutions of tryptophan (B), tyrosine-tryptophan mixture (C) and Peptide M (D) were diluted four fold before measurements due to the high absorbance of the original 1 M solution.

Supplementary Table 1. Resonance assignments of ${}^{1}H$ chemical shifts of Peptide M at pH 5.0 using solution NMR experiments.

Residue	NH (ppm)	CαH (ppm)	CβH (ppm)	Others (ppm)
**I1**	NA	3.882	1.954	NA
**M2**	8.717	4.516	2.021	γ-CH_2 2.558
**D3**	8.556	4.614	2.724	
**R4**	8.357	4.217	1.641	γ-CH_2 1.389
				δ-CH_2 3.081
				NH 7.090
**Y5**	8.113	4.609	3.027/2.905	δ-H 7.049
				ε-H 6.726
**R6**	8.056	4.356	1.735	γ-CH_2 1.538
				δ-CH_2 3.154
				NH 7.191
**V7**	8.160	4.126	2.012	γ-CH_3 0.9080
**R8**	8.485	4.360	1.702/1.795	γ-CH_2 1.568
				δ-CH_2 3.140
				NH 7.191
**N9**	8.585	4.630	2.793	
**G10**	8.440	3.930		
**D11**	8.207	4.650	2.774	
**R12**	8.240	4.237	1.654	γ-CH_2 1.442
				δ-CH_2 3.019
				NH 7.020

**I13**	8.094	4.162	1.775	γ-CH 1.114
				δ-CH_3 0.805
**W14**	8.312	4.695	3.256/3.137	δ-H 7.207
				ε-H 7.581
				ζ2-H 7.457
				ζ3-H 7.167
				ε-NH_3^+ 7.128
**I15**	7.980	4.086	1.731	γ-CH 1.034
				δ-CH_3 0.856
**R16**	8.223	4.237	1.757	γ-CH_2 1.523
				δ-CH_2 3.126
**L17**	8.223	4.339	1.620	γ-CH 0.888
				δ-CH_3 0.841
**R18**	7.973	4.197	1.851	γ-CH_2 1.576
				δ-CH_2 3.167

Supplementary Table 2. Statistics for the 20 lowest energy NMR structures.

Parameter	Value
**Distance and Angle Restraints**	
Total NOEs	161
Intraresidual	73
Interresidual	88
Angles	9
Hydrogen bond restraints	--
Total restraints per residue	8.9
**Statistics for Calculated Structures**	
R.M.S.D. (Å)	
Backbone	0.59 ± 0.17
All Heavy Atoms	1.68 ± 0.31

Supplementary Table 3. NOEs used for calculations

assign (residue 1 and name HA) (residue 1 and name HB#) 3.0 1.2 0.6 !m
assign (residue 1 and name HA) (residue 1 and name HG#) 5.0 2.2 1.0 !w
assign (residue 2 and name HA) (residue 2 and name HB#) 5.0 2.2 1.0 !w
assign (residue 2 and name HA) (residue 2 and name HG#) 5.0 2.2 1.0 !w
assign (residue 2 and name HA) (residue 2 and name HN) 5.0 2.2 1.0 !w
assign (residue 2 and name HA) (residue 3 and name HN) 2.5 0.7 0.4 !s
assign (residue 2 and name HB#) (residue 4 and name HB#) 5.0 2.2 1.0 !w
assign (residue 2 and name HA) (residue 17 and name HG#) 5.0 2.2 1.0 !w
assign (residue 2 and name HA) (residue 17 and name HA) 5.0 2.2 1.0 !w
assign (residue 2 and name HB#) (residue 2 and name HG#) 5.0 2.2 1.0 !w
assign (residue 2 and name HB#) (residue 3 and name HN) 5.0 2.2 1.0 !w
assign (residue 2 and name HG#) (residue 2 and name HN) 5.0 2.2 1.0 !w
assign (residue 3 and name HA) (residue 3 and name HN) 3.0 1.2 0.6 !m
assign (residue 3 and name HA) (residue 4 and name HN) 2.5 0.7 0.4 !s
assign (residue 3 and name HA) (residue 3 and name HB1#) 5.0 2.2 1.0 !w
assign (residue 3 and name HA) (residue 3 and name HB2#) 5.0 2.2 1.0 !w
assign (residue 3 and name HB#) (residue 3 and name HN) 5.0 2.2 1.0 !w
assign (residue 3 and name HA) (residue 4 and name HA) 5.0 2.2 1.0 !w
assign (residue 3 and name HB#) (residue 4 and name HN) 5.0 2.2 1.0 !w
assign (residue 3 and name HB#) (residue 4 and name HN) 5.0 2.2 1.0 !w
assign (residue 3 and name HA) (residue 16 and name HN) 5.0 2.2 1.0 !w
assign (residue 3 and name HB#) (residue 16 and name HD#) 5.0 2.2 1.0 !w
assign (residue 3 and name HA) (residue 16 and name HA) 5.0 2.2 1.0 !w
assign (residue 3 and name HA) (residue 16 and name HD#) 5.0 2.2 1.0 !w
assign (residue 3 and name HB#) (residue 5 and name HE#) 5.0 2.2 1.0 !w
assign (residue 4 and name HA) (residue 5 and name HN) 2.5 0.7 0.4 !s
assign (residue 4 and name HA) (residue 4 and name HD#) 5.0 2.2 1.0 !w
assign (residue 4 and name HA) (residue 4 and name HN) 5.0 2.2 1.0 !w
assign (residue 4 and name HA) (residue 4 and name HB#) 3.0 1.2 0.6 !m
assign (residue 4 and name HD#) (residue 4 and name HB#) 5.0 2.2 1.0 !w
assign (residue 4 and name HD#) (residue 4 and name HG#) 5.0 2.2 1.0 !w
assign (residue 4 and name HG#) (residue 15 and name HG#) 5.0 2.2 1.0 !w
assign (residue 4 and name HB#) (residue 5 and name HN) 5.0 2.2 1.0 !w
assign (residue 4 and name HG#) (residue 15 and name HA#) 5.0 2.2 1.0 !w
assign (residue 4 and name HB#) (residue 15 and name HN) 5.0 2.2 1.0 !w
assign (residue 4 and name HA) (residue 15 and name HB#) 5.0 2.2 1.0 !w
assign (residue 4 and name HG#) (residue 6 and name HG#) 5.0 2.2 1.0 !w
assign (residue 5 and name HA) (residue 6 and name HN) 2.5 0.7 0.4 !s
assign (residue 5 and name HA) (residue 5 and name HB#) 5.0 2.2 1.0 !w
assign (residue 5 and name HA) (residue 5 and name HB#) 5.0 2.2 1.0 !w
assign (residue 5 and name HA) (residue 5 and name HN) 5.0 2.2 1.0 !w
assign (residue 5 and name HA) (residue 5 and name HD#) 5.0 2.2 1.0 !w
assign (residue 5 and name HB#) (residue 6 and name HN) 5.0 2.2 1.0 !w
assign (residue 5 and name HB1) (residue 5 and name HB2) 2.5 0.7 0.4 !s

assign (residue 5 and name HB#) (residue 5 and name HN) 5.0 2.2 1.0 !w
assign (residue 5 and name HB#) (residue 7 and name HG#) 5.0 2.2 1.0 !w
assign (residue 5 and name HA) (residue 5 and name HE#) 5.0 2.2 1.0 !w
assign (residue 5 and name HD#) (residue 14 and name HB#) 5.0 2.2 1.0 !w
assign (residue 5 and name HB#) (residue 14 and name HE#) 5.0 2.2 1.0 !w
assign (residue 5 and name HD#) (residue 5 and name HB#) 5.0 2.2 1.0 !w
assign (residue 5 and name HD#) (residue 14 and name HE#) 5.0 2.2 1.0 !w
assign (residue 5 and name HA) (residue 14 and name HB#) 5.0 2.2 1.0 !w
assign (residue 5 and name HB#) (residue 14 and name HZ#) 5.0 2.2 1.0 !w
assign (residue 5 and name HE#) (residue 14 and name HZ#) 5.0 2.2 1.0 !w
assign (residue 5 and name HE#) (residue 14 and name HD#) 5.0 2.2 1.0 !w
assign (residue 5 and name HB#) (residue 6 and name HN) 5.0 2.2 1.0 !w
assign (residue 6 and name HA) (residue 7 and name HN) 2.5 0.7 0.4 !s
assign (residue 6 and name HA) (residue 13 and name HB#) 5.0 2.2 1.0 !w
assign (residue 6 and name HA) (residue 6 and name HG#) 5.0 2.2 1.0 !w
assign (residue 6 and name HA) (residue 6 and name HB#) 5.0 2.2 1.0 !w
assign (residue 6 and name HA) (residue 6 and name HN) 5.0 2.2 1.0 !w
assign (residue 6 and name HD#) (residue 6 and name HG#) 2.5 0.7 0.4 !s
assign (residue 6 and name HA) (residue 13 and name HA) 5.0 2.2 1.0 !w
assign (residue 6 and name HG#) (residue 13 and name HB#) 5.0 2.2 1.0 !w
assign (residue 6 and name HD#) (residue 13 and name HB#) 5.0 2.2 1.0 !w
assign (residue 6 and name HG#) (residue 4 and name HG#) 5.0 2.2 1.0 !w
assign (residue 7 and name HA) (residue 7 and name HB#) 5.0 2.2 1.0 !w
assign (residue 7 and name HA) (residue 7 and name HN) 5.0 2.2 1.0 !w
assign (residue 7 and name HA) (residue 7 and name HG#) 5.0 2.2 1.0 !w
assign (residue 7 and name HA) (residue 8 and name HN) 2.5 0.7 0.4 !s
assign (residue 7 and name HB#) (residue 8 and name HN) 5.0 2.2 1.0 !w
assign (residue 7 and name HG#) (residue 7 and name HB#) 3.0 1.2 0.6 !m
assign (residue 7 and name HG#) (residue 8 and name HN) 5.0 2.2 1.0 !w
assign (residue 8 and name HA) (residue 8 and name HN) 2.5 0.7 0.4 !s
assign (residue 8 and name HA) (residue 9 and name HN) 3.0 1.2 0.6 !m
assign (residue 8 and name HB#) (residue 9 and name HN) 5.0 2.2 0.6 !w
assign (residue 8 and name HB#) (residue 6 and name HG#) 5.0 2.2 0.6 !w
assign (residue 8 and name HD#) (residue 8 and name HB#) 5.0 2.2 0.6 !m
assign (residue 8 and name HA) (residue 8 and name HB#) 5.0 2.2 1.0 !w
assign (residue 8 and name HA) (residue 8 and name HB#) 5.0 2.2 1.0 !w
assign (residue 9 and name HA) (residue 9 and name HN) 3.0 1.2 0.6 !m
assign (residue 9 and name HA) (residue 10 and name HN) 3.0 1.2 0.6 !m
assign (residue 9 and name HA) (residue 9 and name HB#) 5.0 2.2 1.0 !w
assign (residue 9 and name HB#) (residue 9 and name HN) 5.0 2.2 1.0 !w
assign (residue 9 and name HB#) (residue 10 and name HN) 5.0 2.2 1.0 !w
assign (residue 10 and name HA#) (residue 10 and name HN) 3.0 1.2 0.6 !m
assign (residue 10 and name HA#) (residue 11 and name HN) 2.5 0.7 0.4 !s
assign (residue 11 and name HA) (residue 12 and name HN) 2.5 0.7 0.4 !s
assign (residue 11 and name HA) (residue 11 and name HN) 5.0 2.2 1.0 !w
assign (residue 11 and name HA) (residue 11 and name HB#) 3.0 1.2 0.6 !m

assign (residue 11 and name HB#) (residue 11 and name HN) 3.0 1.2 0.6 !m
assign (residue 11 and name HA) (residue 12 and name HN) 5.0 2.2 1.0 !w
assign (residue 11 and name HB#) (residue 8 and name HG#) 5.0 2.2 1.0 !w
assign (residue 11 and name HB#) (residue 13 and name HB#) 5.0 2.2 1.0 !w
assign (residue 12 and name HA) (residue 13 and name HN) 2.5 0.7 0.4 !s
assign (residue 12 and name HA) (residue 12 and name HB#) 5.0 2.2 1.0 !w
assign (residue 12 and name HA) (residue 12 and name HD#) 5.0 2.2 1.0 !w
assign (residue 12 and name HA) (residue 12 and name HN) 5.0 2.2 1.0 !w
assign (residue 12 and name HB#) (residue 7 and name HG#) 5.0 2.2 1.0 !w
assign (residue 12 and name HD#) (residue 7 and name HG#) 5.0 2.2 1.0 !w
assign (residue 12 and name HD#) (residue 7 and name HB#) 5.0 2.2 1.0 !w
assign (residue 12 and name HG#) (residue 7 and name HG#) 5.0 2.2 1.0 !w
assign (residue 12 and name HB#) (residue 13 and name HN) 5.0 2.2 1.0 !w
assign (residue 12 and name HD#) (residue 14 and name HZ#) 5.0 2.2 1.0 !w
assign (residue 13 and name HA) (residue 14 and name HN) 2.5 0.7 0.4 !s
assign (residue 13 and name HA) (residue 13 and name HB#) 5.0 2.2 1.0 !w
assign (residue 13 and name HA) (residue 13 and name HG#) 5.0 2.2 1.0 !w
assign (residue 13 and name HA) (residue 13 and name HN) 5.0 2.2 1.0 !w
assign (residue 13 and name HA) (residue 14 and name HN) 2.5 0.7 0.4 !s
assign (residue 13 and name HB#) (residue 13 and name HN) 2.5 0.7 0.4 !s
assign (residue 13 and name HA) (residue 14 and name HZ#) 5.0 2.2 1.0 !w
assign (residue 13 and name HG#) (residue 13 and name HN) 5.0 2.2 1.0 !w
assign (residue 13 and name HB#) (residue 14 and name HN) 5.0 2.2 1.0 !w
assign (residue 14 and name HA) (residue 15 and name HN) 2.5 0.7 0.4 !s
assign (residue 14 and name HA) (residue 14 and name HB#) 5.0 2.2 1.0 !w
assign (residue 14 and name HB#) (residue 14 and name HE#) 5.0 2.2 1.0 !w
assign (residue 14 and name HB#) (residue 14 and name HZ#) 5.0 2.2 1.0 !w
assign (residue 15 and name HA) (residue 16 and name HN) 2.5 0.7 0.4 !s
assign (residue 15 and name HA) (residue 15 and name HB#) 5.0 2.2 1.0 !w
assign (residue 15 and name HA) (residue 15 and name HD#) 5.0 2.2 1.0 !w
assign (residue 15 and name HA) (residue 15 and name HN) 5.0 2.2 1.0 !w
assign (residue 15 and name HB#) (residue 15 and name HG#) 5.0 2.2 1.0 !w
assign (residue 15 and name HB#) (residue 15 and name HD#) 3.0 1.2 0.6 !m
assign (residue 15 and name HG#) (residue 15 and name HD#) 3.0 1.2 0.6 !m
assign (residue 15 and name HG#) (residue 14 and name HN) 5.0 2.2 1.0 !w
assign (residue 15 and name HB#) (residue 17 and name HD#) 5.0 2.2 1.0 !w
assign (residue 16 and name HA) (residue 16 and name HN) 2.5 0.7 0.4 !s
assign (residue 16 and name HA) (residue 17 and name HN) 2.5 0.7 0.4 !s
assign (residue 16 and name HD#) (residue 16 and name HB#) 5.0 2.2 1.0 !w
assign (residue 16 and name HB#) (residue 17 and name HN) 5.0 2.2 1.0 !w
assign (residue 16 and name HG#) (residue 18 and name HB#) 5.0 2.2 1.0 !w
assign (residue 17 and name HA) (residue 17 and name HN) 5.0 2.2 1.0 !w
assign (residue 17 and name HA) (residue 17 and name HB#) 3.0 1.2 0.6 !m
assign (residue 17 and name HA) (residue 17 and name HD#) 3.0 1.2 0.6 !m
assign (residue 17 and name HA) (residue 18 and name HN) 2.5 0.7 0.4 !s
assign (residue 17 and name HD#) (residue 15 and name HG#) 5.0 2.2 1.0 !w

assign (residue 17 and name HD#) (residue 17 and name HB#) 3.0 1.2 0.6 !m
assign (residue 18 and name HA) (residue 18 and name HN) 3.0 1.2 0.6 !m
assign (residue 18 and name HD#) (residue 16 and name HG#) 5.0 2.2 1.0 !w
assign (residue 18 and name HA) (residue 18 and name HB#) 5.0 2.2 1.0 !w
assign (residue 18 and name HA) (residue 18 and name HB#) 5.0 2.2 1.0 !w
assign (residue 18 and name HA) (residue 18 and name HD#) 5.0 2.2 1.0 !w
assign (residue 18 and name HB#) (residue 18 and name HD#) 5.0 2.2 1.0 !w
assign (residue 18 and name HA) (residue 18 and name HG#) 5.0 2.2 1.0 !w
assign (residue 18 and name HA) (residue 2 and name HA) 2.5 0.7 0.4 !s
! NH-NH
assign (residue 2 and name HN) (residue 3 and name HN) 5.0 2.2 1.0 !w
assign (residue 3 and name HN) (residue 4 and name HN) 5.0 2.2 1.0 !w
assign (residue 4 and name HN) (residue 5 and name HN) 5.0 2.2 1.0 !w
assign (residue 5 and name HN) (residue 6 and name HN) 5.0 2.2 1.0 !w
assign (residue 7 and name HN) (residue 8 and name HN) 5.0 2.2 1.0 !w
assign (residue 9 and name HN) (residue 10 and name HN) 5.0 2.2 1.0 !w
assign (residue 10 and name HN) (residue 11 and name HN) 5.0 2.2 1.0 !w
assign (residue 11 and name HN) (residue 12 and name HN) 5.0 2.2 1.0 !w
assign (residue 12 and name HN) (residue 13 and name HN) 5.0 2.2 1.0 !w
assign (residue 13 and name HN) (residue 14 and name HN) 5.0 2.2 1.0 !w
assign (residue 14 and name HN) (residue 15 and name HN) 5.0 2.2 1.0 !w
assign (residue 15 and name HN) (residue 16 and name HN) 5.0 2.2 1.0 !w
assign (residue 16 and name HN) (residue 17 and name HN) 5.0 2.2 1.0 !w
assign (residue 17 and name HN) (residue 18 and name HN) 5.0 2.2 1.0 !w
assign (residue 4 and name HN) (residue 15 and name HN) 5.0 2.2 1.0 !w
assign (residue 3 and name HN) (residue 16 and name HN) 5.0 2.2 1.0 !w

Supplementary Table 4. Distances (Å) derived from the 20 lowest energy structural models and the averaged, minimized NMR structure of Peptide M

**MODEL #**	**Y5O-W14NH**	**Y5O-R16NεH**	**Y5OH-R16Nε**	**Y5O-R16NH***	**Y5OH-R16N***
**1**	6.1	9.3	9.7	9.7	10.9
**2**	5.9	6.4	7.2	8.2	9.2
**3**	6.2	5.4	5.2	1.5	3.1
**4**	6.0	9.3	9.7	7.9	9.6
**5**	6.4	2.8	3.7	1.4	3.1
**6**	6.3	4.2	4.2	1.5	3.1
**7**	6.3	8.5	6.9	5.5	5.4
**8**	6.5	5.1	5.3	1.4	3.2
**9**	6.7	5.7	4.2	3.7	3.3
**10**	6.2	4.9	4.4	2.9	2.9
**11**	6.5	5.1	4.8	7.3	6.8
**12**	6.4	7.0	6.0	7.4	7.0
**13**	6.4	8.4	7.5	9.5	9.0
**14**	6.9	7.8	8.0	7.7	8.8
**15**	6.7	4.8	4.4	2.7	2.8
**16**	6.7	6.5	6.1	5	5.3
**17**	6.0	9.2	7.6	7.1	6.5
**18**	6.6	6.9	5.7	5.7	5.8
**19**	6.6	4.9	5.1	1.4	3.1
**20**	6.3	6.7	6.0	7.3	7.1
**AVE, MIN.**	**6.4**	**8.2**	**6.8**	**6.3**	**5.5**

*For simplicity, only the distance to the closest–NH group (η^1 or η^2) of R16 is presented.

Supplementary Table 5. Distances (Å) derived from the 20 lowest energy structural models and the averaged, minimized NMR structure of Peptide A[1]

**MODEL #**	Y5O-H14NH	Y5**O**-R16**NεH**	Y5**OH**-R16**Nε**	Y5O-R16**NH***	Y5**OH**-R16**N***
**1**	3.8	1.8	2.8	3.8	3.8
**2**	3.8	1.8	2.8	3.9	3.8
**3**	3.8	1.9	2.6	4.0	3.7
**4**	2.2	5.6	5.3	6.2	5.9
**5**	4.0	1.5	3.0	4.0	4.1
**6**	2.3	5.6	5.2	5.8	6.0
**7**	2.3	5.6	5.2	6.0	6.2
**8**	2.1	4.3	5.1	3.8	3.6
**9**	3.7	3.9	4.1	1.4	3.1
**10**	2.3	5.6	5.3	6.1	6.3
**11**	2.2	5.6	5.3	6.2	6.3
**12**	3.8	4.4	3.4	1.5	2.6
**13**	2.5	4.8	4.9	3.0	4.3
**14**	3.2	5.5	5.1	5.9	6.0
**15**	3.3	5.5	4.0	5.8	5.9
**16**	2.4	4.0	4.0	1.5	2.9
**17**	2.5	4.0	3.4	1.5	2.9
**18**	3.8	4.1	4.3	1.5	3.0
**19**	2.1	5.1	4.3	3.8	3.7
**20**	3.8	3.0	3.1	1.4	4.1
**AVE, MIN.**	**4.0**	**1.6**	**2.9**	**3.8**	**3.9**

*For simplicity, only the distance to the closest–NH group (η^1 or η^2) of R16 is presented.

Supplementary References

1. Sibert, R. *et al.* Proton-coupled electron transfer in a biomimetic peptide as a model of enzyme regulatory mechanisms. *J. Am. Chem. Soc.* **129**, 4393-4400 (2007).

2. Vassiliev, I. R., Offenbacher, A. R. & Barry, B. A. Redox-active tyrosine residues in pentapeptides. *J. Phys. Chem. B* **109**, 23077-23085 (2005).

3. Offenbacher, A. R., Vassiliev, I. R., Seyedsayamdost, M. R., Stubbe, J. & Barry, B. A. Redox-linked structural changes in ribonucleotide reductase. *J. Am. Chem. Soc.* **131**, 7496-7497 (2009).
